# Spirulina and Its Bioactive Compounds as Multi-Target Anticancer Agents: Mechanisms, Immune Modulation, and Translational Potential

**DOI:** 10.3390/medsci14020189

**Published:** 2026-04-10

**Authors:** Rym Akrout, Khouloud Ayed, Hela Mrizak, Ludovic Leloup, Orace Mathieu Kenou, Fidèle Fassinou, Dhouha Bacha, Rahma Boughriba, Hanen Attia, Hervé Kovacic, Wassim Y. Almawi, Asma Gati

**Affiliations:** 1 Laboratory of Genetics Immunology and Human Pathology, Biology Department, Faculty of Sciences of Tunis, University of Tunis El Manar, Tunis 2092, Tunisia; 2 Pharmacology Unit, School of Pharmacy, Center for Neuroscience, University of Camerino, 62032 Camerino, Italy; 3 Faculté des Sciences Médicales et Paramédicales, Institut de NeuroPhysiopathologie (INP), UMR 7051, CNRS, Aix Marseille Université, 13005 Marseille, France; 4 Laboratoire de Biologie Intégrative pour l’Innovation Thérapeutique (BioInov), Faculté des Sciences et Techniques (FAST), Université d’Abomey-Calavi, Abomey-Calavi BP 526, Benin; 5 Faculty of Sciences, University Campus at El-Manar, Tunis 2092, Tunisia

**Keywords:** anticancer mechanisms, chemoprotection, immunomodulation, natural products, Spirulina

## Abstract

Marine-derived natural products are increasingly recognized for their therapeutic potential in cancer and other chronic diseases. Despite significant advances, current cancer treatments remain challenged by toxicity, drug resistance, and limited survival benefits. Natural compounds offer promising alternatives due to their multi-target mechanisms and favorable safety profiles. Among them, Spirulina, a filamentous cyanobacterium, stands out for its rich composition and diverse biological activities. Its anticancer effects involve apoptosis induction via intrinsic and extrinsic pathways, cell cycle arrest at G1/S or G2/M phases, inhibition of angiogenesis through the VEGF/VEGFR2 axis, and suppression of epithelial–mesenchymal transition. These activities are mainly attributed to C-phycocyanin, allophycocyanin, phenolic compounds, and immunomodulatory polysaccharides. Spirulina also exhibits potent immunomodulatory effects by enhancing natural killer cell activity, promoting M1 macrophage polarization, and regulating Th1 and Th17 cytokine responses, highlighting its potential as both an immunotherapeutic and chemoprotective agent. Moreover, preclinical findings suggest it may reduce chemotherapy-associated side effects. However, translation into clinical therapy remains limited by low bioavailability, lack of standardized extracts, and scarce clinical evidence. This review summarizes current mechanistic and immunological insights and highlights the need for optimized formulations, defined dosing strategies, and well-designed clinical trials to validate Spirulina’s potential in cancer treatment.

## 1. Introduction

Cancer remains the second leading cause of death worldwide, after cardiovascular disease, and continues to be a major public health issue. According to GLOBOCAN 2022, there were approximately 19.3 million new cancer cases and 10 million cancer-related deaths globally [1]. Despite advances in prevention, early detection, and treatment technologies, global cancer incidence and mortality have shown little overall improvement over the past thirty years [2]. Standard treatments, such as surgery, radiotherapy, chemotherapy, and immunotherapy, often yield less-than-ideal results. For instance, chemotherapy achieves complete response rates of only 7–11% in advanced solid tumors [3]. Moreover, metastasis accounts for 90% of cancer deaths and remains largely incurable [4]. Treatment-related toxicities affect up to 86% of patients, with 27% experiencing severe or life-threatening adverse effects [5]. Drug resistance is nearly universal in advanced cancers, driven by tumor heterogeneity, adaptive cellular mechanisms, and the persistence of cancer stem cells [6]. In addition, the economic burden is substantial, with cancer care costs exceeding $200 billion annually in the United States (U.S.) alone [7].

These limitations of standard therapies underscore the urgent need for alternative strategies capable of targeting multiple oncogenic pathways while maintaining an acceptable safety profile. Natural products have played a pivotal role in this regard, contributing to nearly 60% of all approved anticancer agents, including paclitaxel, vincristine, and doxorubicin [8]. Marine-derived compounds have garnered special interest due to their unique structures and strong biological activity, often working through different mechanisms than those from land-based sources [9]. Their high heteroatom content, particularly bromine, appears to enhance their biological activity [10,11]. In medicinal chemistry, halogens are often introduced to improve membrane permeability and support the movement of active molecules across cell membranes and the blood–brain barrier [12].

**Figure 1:**
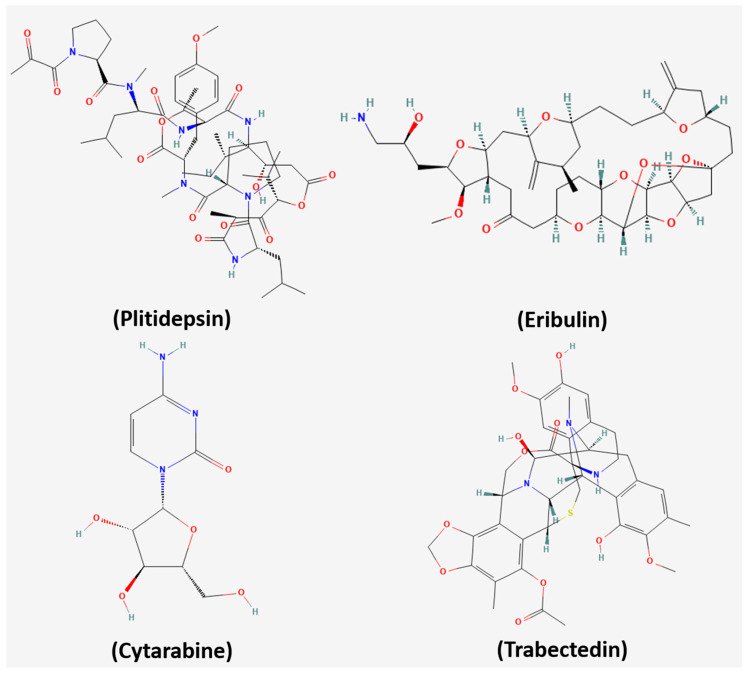
Chemical structures of selected marine-derived anticancer agents in clinical trials. The structures of plitidepsin, eribulin mesylate, cytarabine, and trabectedin were obtained from PubChem.

To date, 34 marine-derived substances have reached clinical trials [13], and nine drugs of marine origin, including enfortumab vedotin, beamtimes mafodotin, plitidepsin, polatuzumab vedotin, eribulin mesylate, brentuximab vedotin, cytarabine, and trabectedin, have received Food and Drug Administration (FDA) approval (Figure 1) [14]. These developments highlight the translational potential of marine bioactive molecules and underscore the need for further studies to fully exploit their therapeutic properties.

Among marine-derived resources, the filamentous cyanobacterium Spirulina is gaining increasing attention for its exceptional nutritional profile and diverse pharmacological activities. Unlike many marine bioactive compounds that require labor-intensive extraction or chemical synthesis, Spirulina can be cultivated efficiently and at low cost, highlighting its scalability for therapeutic use [15]. It contains over 100 biologically active compounds and has been granted the “Generally Recognized as Safe” (GRAS) status by the U.S. FDA, owing to its long history of safe consumption in different cultures [16]. Collectively, this makes Spirulina a promising, accessible candidate for integrative oncology and broader clinical applications.

Although promising preclinical evidence supports the anticancer potential of Spirulina, significant knowledge gaps still exist, impeding its translation into clinical practice. Most published reviews have narrowly focused on individual bioactive components or specific cancer models without providing a comprehensive mechanistic framework or assessing the feasibility of clinical application [17,18]. Furthermore, variations in experimental protocols, inconsistent extract formulations, and the lack of standardized dose–response evaluations have complicated the interpretation and reproducibility of results. In particular, the disconnect between in vitro and in vivo findings, as well as the limited scope of clinical validation, remains insufficiently addressed in the current literature, emphasizing the urgent need for translational research.

## 2. Rationale and Unique Contribution of This Review

This review offers an integrated synthesis of the current evidence on Spirulina’s anticancer potential, highlighting its perception from a traditional nutraceutical to a multi-target therapeutic candidate in oncology. Earlier reviews have addressed aspects of Spirulina’s anticancer activity, but the literature remains fragmented. A unified assessment linking its major bioactive compounds with mechanistic pathways, preclinical evidence, and clinical findings has been missing. This review fills that gap by outlining Spirulina’s influence on key cancer-related processes, including apoptosis, oxidative stress, proliferation, angiogenesis, metastasis, and immune modulation, and by examining its potential to reduce chemotherapy-related toxicity. It also discusses major translational challenges, such as bioavailability, pharmacokinetics, clinical limitations, and regulatory barriers. By connecting natural product research with principles of precision oncology, this review offers novel insights into Spirulina as a potential adjunct in cancer prevention and therapy, informing future strategies for integrative cancer treatment.

## 3. Literature Search and Selection Criteria

We conducted a systematic search of PubMed, Scopus, Web of Science, ScienceDirect, and Google Scholar to identify studies examining Spirulina and its bioactive compounds as multi-target anticancer agents. Search terms included “Spirulina,” “Arthrospira platensis,” “C-phycocyanin,” “bioactive compounds,” “cancer,” “apoptosis,” “oxidative stress,” “angiogenesis,” “metastasis,” “immunomodulatory effects,” “chemotherapy,” “chemoprotection,” “bioavailability,” and “pharmacokinetics,” combined with Boolean operators.

Articles published in English from 1985 to 2025 were considered. Preprints, non-English articles, and studies without experimental or mechanistic data were excluded. The review draws on in vitro, in vivo, and clinical research, along with systematic reviews and meta-analyses, focusing on anticancer activity, immune effects, chemoprotection, and translational relevance.

## 4. Chemical Composition and Anticancer Relevance of Spirulina Bioactive Compounds

### 4.1. Phycobiliproteins: Primary Anticancer Effectors

Phycobiliproteins are among the most thoroughly researched anticancer components of Spirulina, constituting 15–25% of its total protein content [19]. These water-soluble, fluorescent proteins are involved in photosynthesis and have various biological effects, including triggering apoptosis, regulating cell cycle progression, and modulating immune responses [20]. Their amphiphilic nature, structural stability, bioavailability, and low toxicity make them promising options for therapeutic development [21].

C-phycocyanin (C-PC) is the main anticancer phycobiliprotein, making up 10–20% of Spirulina’s dry weight, with concentrations of 140–200 mg/g in optimized cultures [22]. C-PC (Mr ~83 kDa) [23] consists of α (162 aa) and β (172 aa) subunits, each covalently attached to phycocyanobilin chromophores [24]. It forms stable (αβ)_3_ trimers and (αβ)_6_ hexamers, which are essential for its biological activity [25].

Allophycocyanin exhibits anticancer properties distinct from C-PC [26,27]. It acts synergistically with C-PC to enhance apoptosis, boost immune activation, and improve bioavailability, showing notable activity against hormone-dependent cancers [28].

Phycoerythrin: The presence of the third phycobiliprotein, phycoerythrin (PE), in Spirulina remains uncertain. Some studies report low levels of PE, while others have not detected it at all, and its functional role in Spirulina continues to be investigated [29].

### 4.2. Chlorophylls and Photosynthetic Pigments

**Figure 2:**
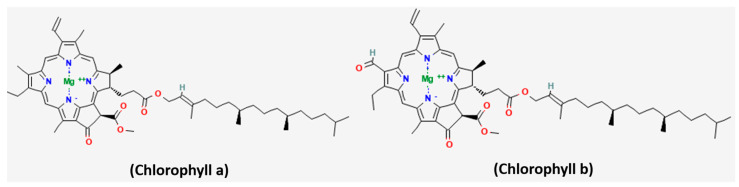
Chemical structures of Chlorophyll a and Chlorophyll b. Structures were obtained from PubChem.

Spirulina contains high levels of chlorophylls, with chlorophyll a (3.29 mg/g) occurring in greater abundance than chlorophyll b (1.17 mg/g) (Figure 2) [30]. Evidence shows that chlorophylls possess chemopreventive properties, which are linked to the activation of Phase II detoxifying enzymes, thereby strengthening cellular defense against oxidative stress [31,32]. Remarkably, chlorophyll a works synergistically with phycobiliproteins, enhancing cellular uptake and boosting anticancer effects [18,33]. This underscores the multifunctional role of chlorophylls in Spirulina, highlighting their therapeutic and preventive potential within integrative anticancer strategies.

### 4.3. Carotenoids and Antioxidant Pigments

**Figure 3:**
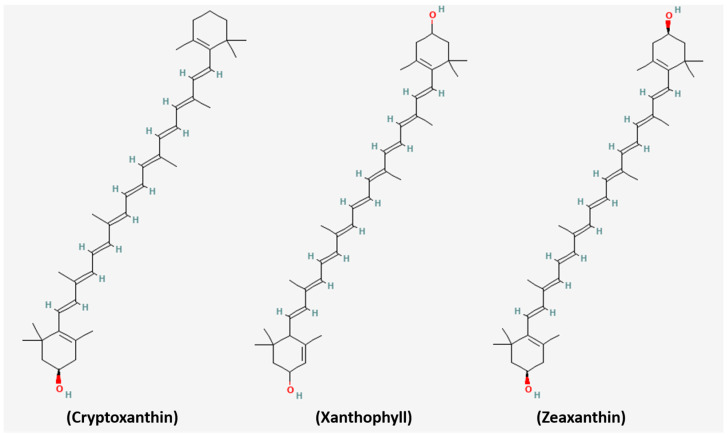
Chemical structures of Cryptoxanthin, Xanthophyll and Zeaxanthin. Structures were obtained from PubChem.

Spirulina provides a rich source of carotenoids, particularly β-carotene (700–1700 mg/kg) [34], which is roughly tenfold higher than in carrots [35]. β-Carotene exhibits strong antioxidant properties, which reportedly enable the prevention of DNA damage [36], enhance natural killer (NK) cell-mediated immune surveillance [37,38], facilitate gap junction communication to slow tumor growth, and regulate the activity of NF-κB, AP-1, and other transcription factors [39,40]. Other carotenoids include Zeaxanthin (58–80 mg/100 g) [41], β-cryptoxanthin (6.30–10 mg/100 g) [42], and Xanthophylls (20–170 mg/100 g) [43], which work together to enhance anticancer and photoprotective effects (Figure 3) [44]. Clinically, Spirulina-derived β-carotene effectively increases total-body vitamin A stores [45], supporting its role in cancer prevention, eye health, and as an additional antioxidant in managing chronic diseases [44,46].

### 4.4. Bioactive Polysaccharides and Immunomodulatory Components

Spirulina polysaccharides (PSP) make up 10–20% of its dry weight [47] and mainly consist of glucose, galactose, xylose, rhamnose, mannose and fucose, with their molar ratios often influenced by the source material and extraction method [48]. Multiple studies have shown that the immunostimulatory effects of PSP are strongly associated with their molecular weight, with PSP ranging from 10 to 1000 kDa exhibiting the most potent immunomodulatory activity [49]. These effects include enhanced NK cell cytotoxicity (up to 130%) [50], macrophage activation via the Toll-Like Receptor 4 (TLR4) pathway [51], and stimulation of monocytes through CD14 and TLR2 [52].

Lipopolysaccharides (LPS) make up 1.0–1.6% of Spirulina’s dry weight [53]. Unlike LPS from Gram-negative bacteria, which can trigger severe inflammation and septic shock [54], Spirulina LPS has a markedly lower inflammatory potential due to its unique fatty acid composition, including 3-hydroxy myristate and branched-chain fatty acids [55]. These structural features allow immune activation without harmful systemic effects, stimulating both innate and adaptive immunity via antigen-presenting cells. Spirulina LPS shows promise as a safe vaccine adjuvant and immunotherapy enhancer, improving antibody responses and immune surveillance without provoking toxic inflammatory cascades [56,57,58].

### 4.5. Phenolic Compounds and Secondary Metabolites

**Figure 4:**
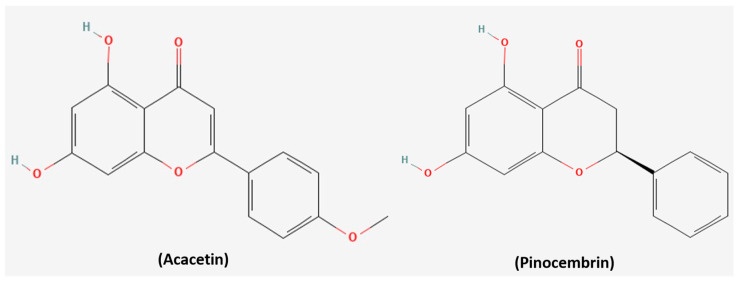
Chemical structures of Acacetin and Pinocembrin. Structures were obtained from PubChem Collectively, these Spirulina-derived compounds form the foundation of its anticancer activity. The next section links their composition with functional evidence, demonstrating how Spirulina and its constituents influence redox balance, cell-death pathways, proliferation, angiogenesis, and EMT to connect chemical features with biological effects.

Phenolic compounds, including flavonoids, phenolic acids, and complex polyphenols, constitute an important class of secondary metabolites present in Spirulina [59]. The main constituents are Acacetin (53.6% of total phenolics, 35.37 µg/mg) and Pinocembrin (41.28%, 27.23 µg/mg) (Figure 4) [60]. Extraction methods strongly influence phenolic yield, with supercritical CO_2_ extraction producing up to three times higher concentrations than traditional solvent-based techniques [61]. These compounds exhibit anticancer effects through potent radical scavenging, transition-metal chelation to inhibit Fenton reactions, and suppression of pro-inflammatory enzymes such as COX-2 and lipoxygenase [62]. Clinically, they may play roles in chemoprevention by protecting cells from DNA damage and reducing oxidative stress–driven cancer development [59]. Certain phenolic compounds can also induce apoptosis and inhibit cancer cell proliferation, underscoring Spirulina’s potential as a complementary cancer therapy [59]. Moreover, phenolic compounds enhance the cellular uptake of carotenoids by upregulating their membrane transporters [63], which could improve the intracellular delivery and efficacy of other bioactive compounds. This may also result in synergistic effects with phycobiliproteins, further enhancing both antioxidant and therapeutic activities.

## 5. Molecular Mechanisms of Spirulina-Mediated Anticancer Activity

### 5.1. Spirulina’s Context-Dependent Pro-Oxidant and Antioxidant Effects

**Figure 5:**
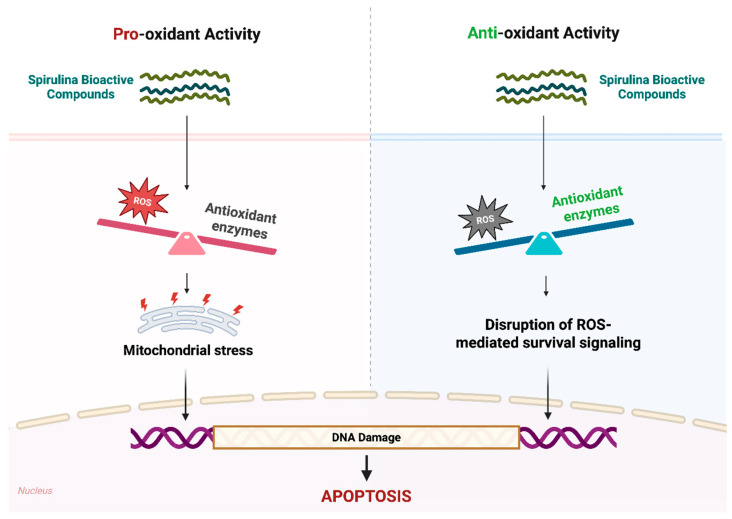
Dual redox-modulating activity of Spirulina bioactive compounds in cancer cells. Spirulina compounds can act as pro-oxidants, increasing ROS and decreasing antioxidant enzymes, or as antioxidants, boosting enzymes like CAT, GPx, GSH, and MnSOD relative to ROS. Both actions lead to DNA damage and apoptosis, exploiting oxidative vulnerabilities to selectively target cancer cells.

Spirulina exhibits a dual redox-modulating capacity, acting as either an antioxidant or a pro-oxidant depending on the cellular context (Figure 5). This dual behavior reflects the redox imbalance inherent in cancer cells, which exhibit higher basal ROS levels and weaker antioxidant defenses than normal cells [64]. Components of Spirulina induce mitochondrial dysfunction, DNA damage, and apoptosis when they raise ROS above a critical threshold. For example, in esophageal adenocarcinoma cells (SK-GT-4), phenolic-rich Spirulina extracts containing gallic acid (52.9 µg/mL), vanillic acid (14.58 µg/mL), syringic acid (12.5 µg/mL), ferulic acid (74.5 µg/mL), and cinnamic acid (36.9 µg/mL) enhanced ROS production, leading to DNA fragmentation and induction of apoptosis [65]. Similarly, treating colon carcinoma cells (Caco-2) with Spirulina (1.25–5% *v*/*v*, 72 h) increased nitric oxide (NO) and ROS production, while superoxide dismutase (SOD) levels decreased by approximately 50%, leading to impaired mitochondrial function and an increased Bax/Bcl-2 ratio [66]. C-PC (30 µM) also triggers apoptosis in rat histiocytic tumor cells (AK-5) by increasing intracellular ROS, leading to Bcl-2 downregulation and caspase-3 activation [67]. Consistently, the Glutathione oxido-reductase-derived peptide from Spirulina (GM15) elevated intracellular ROS at higher concentrations (25 μM, 4 h) in oral cancer (KB) cells, promoting caspase-9 activation and DNA degradation. At a lower concentration (6.25 μM, 4 h), GM15 reduced oxidative stress [68], illustrating a dose-dependent effect in which lower concentrations act as antioxidants, while higher concentrations promote ROS accumulation and oxidative stress. The magnitude of these effects is further shaped by cell-specific features such as mitochondrial activity, antioxidant enzyme expression, iron availability, and oncogenic signaling [69,70].

Conversely, Spirulina can act as an antioxidant, particularly in contexts of excessive oxidative stress. In mice bearing Ehrlich ascites carcinoma, administration of Spirulina nano-emulsion (3225 µg/mL/day, for 2 weeks) to mice bearing Ehrlich ascites carcinoma reduced malondialdehyde (MDA) levels and increased catalase (CAT) activity, effects associated with inhibition of tumor growth [71]. Moreover, Spirulina and its tetrapyrrolic components, including chlorophyllin (125 μM, 24 h) and phycocyanobilin (60 µΜ, 24 h), suppressed pancreatic cancer cell proliferation, partly by depleting intramitochondrial ROS. In the same study, chlorophyllin (30 μM, 24 h) also interfered with the heme catabolic pathway via HMOX, a key effector of the cellular antioxidant defense system [18]. Similarly, in DMBA-induced mammary cancer rats, Spirulina (500 mg/kg/day, for 6 weeks) alleviated oxidative stress by enhancing antioxidant enzymes (SOD, GPx, GSH) and reducing NO and MDA, while inhibiting the Akt/mTOR pathway and activating caspase-3, thereby suppressing tumor growth [70]. Our recent study in glioblastoma cells (U87 and U87-EGFRvIII) further illustrates this context dependence: C-PC (100 µg/mL, 24 h) enhanced cisplatin cytotoxicity in sensitive U87 cells by promoting apoptosis while reducing intracellular ROS and upregulating catalase and MnSOD. In CDDP-resistant U87-EGFRvIII cells, C-PC alone induced apoptosis despite reduced oxidative stress, highlighting that Spirulina components can trigger cell death through redox modulation tailored to the cellular oxidative state [72].

Taken together, these findings indicate that Spirulina-derived compounds do not exert their effects individually as antioxidants or pro-oxidants but as context-dependent redox modulators. The direction and extent of this shift depend on the compound concentration, baseline oxidative state, cell type, and specific experimental conditions. Future studies should evaluate the baseline redox status of different cancer models and apply defined concentrations of Spirulina-derived compounds under standardized conditions. This is crucial for clarifying the mechanisms underlying context-dependent redox modulation and for identifying redox vulnerabilities that may be exploited in cancer therapy.

### 5.2. Mechanisms of Induction of Apoptosis by Spirulina Compounds

Apoptosis is a tightly controlled process crucial for maintaining tissue homeostasis by removing damaged, senescent, or potentially harmful cells. Disruption of apoptotic signaling in cancer is a central pathogenic mechanism, as it allows for uncontrolled cell proliferation, accumulation of genetic mutations, and resistance to treatment [73]. Both extrinsic and intrinsic pathways mediate apoptosis. The extrinsic pathway is activated upon the binding of cellular death ligands, including FasL and TNF-α, to their specific cell-surface receptors, resulting in the recruitment of adaptor proteins and activation of initiator caspases, in particular caspase-8 [73]. On the other hand, the intrinsic pathway is triggered by mitochondrial outer membrane permeabilization (MOMP), leading to the release of cytochrome c and the activation of the apoptosome, which in turn activates caspase-9 [74]. Both pathways ultimately converge on the activation of executioner caspase-3, which cleaves structural and regulatory proteins, leading to the characteristic morphological changes in apoptosis [75]. This includes cell shrinkage, chromatin condensation, and membrane blebbing.

**Figure 6:**
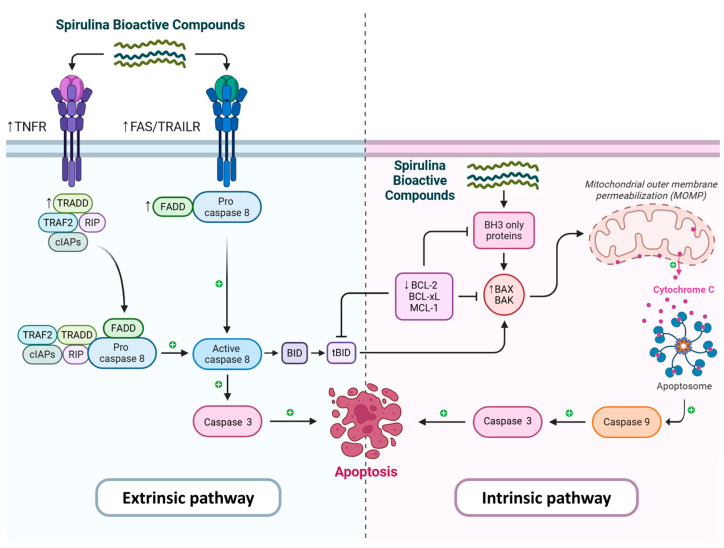
**Apoptosis induction by Spirulina bioactive compounds in cancer cells.** Spirulina compounds selectively trigger apoptosis via intrinsic and extrinsic pathways. The intrinsic pathway involves Bcl-2 family modulation, cytochrome c release, apoptosome assembly, and caspase-9 activation, while the extrinsic pathway involves upregulation of death receptors (FAS, TRAILR, TNFR1), DISC formation, and caspase-8 activation. Both pathways converge on caspase-3, resulting in cancer cell death.

Bioactive compounds derived from Spirulina have been shown to selectively induce apoptosis in cancer cells [76]. Depending on the cancer type, concentration, and exposure time, Spirulina-induced apoptosis is achieved through the following mechanisms (Figure 6):

- **Intrinsic (Mitochondrial) Pathway**: C-PC (5–50 µM, 6–48 h) modulates proteins within the Bcl-2 family, leading to an increase in Bax and a decrease in Bcl-2 expression [77,78,79]. This shift promotes cytochrome c release from mitochondria, leading to apoptosome assembly and caspase-9 activation, which in turn triggers the downstream caspase cascade, including caspase-3, and induces mitochondrial permeability transition pore opening [78].
- **Extrinsic (Death Receptor, DR) Pathway**: Spirulina compounds enhance apoptosis by upregulating death receptors, promoting death-inducing signaling complex (DISC) formation, and activating caspase-8 [79,80]. This pathway can synergize with the intrinsic pathway to amplify apoptotic signaling [80]. C-PC increases Fas receptor (CD95) expression in breast and cervical cancer cells, heightening apoptotic susceptibility [81,82]. In vivo, Spirulina administered at 50–500 mg/kg for 28 days elevates TNFR1, FADD, and TRADD expression in tumor tissues, supporting activation of the extrinsic apoptotic pathway [83]. Additionally, *Spirulina maxima* cultured in deep-sea water enhanced TRAIL-induced apoptosis over seawater-grown strains, which was linked to higher β-carotene and ascorbic acid levels [84].

Collectively, these actions allow Spirulina compounds to preferentially target malignant cells for programmed cell death, supporting their potential use as adjuncts in cancer therapy.

Spirulina also induces autophagy (type II programmed cell death), a catabolic process responsible for degrading oncogenic proteins, misfolded proteins, and damaged organelles. This activity helps maintain cellular homeostasis and suppress tumor development [85]. However, autophagy can exert either cytoprotective or cytotoxic effects depending on the cellular context and the magnitude of autophagic activation. In cancer cells, moderate autophagy may initially function as an adaptive survival response to metabolic or therapeutic stress. In contrast, sustained or excessive autophagic activation can exceed the adaptive capacity of the cell and promote autophagic cell death, particularly in tumors that exhibit resistance to apoptosis [86]. Consistent with this mechanism, C-PC (10 μM, 48 h) has been shown to induce autophagic cell death in pancreatic cancer cells, characterized by elevated Beclin-1 expression and enhanced autophagosome formation. This effect occurs through the suppression of the PI3K/Akt/mTOR and Akt/mTOR/p70S6K signaling pathways, coupled with the activation of the NF-κB pathway, ultimately promoting sustained autophagic flux [87]. Similarly, in non-small-cell lung cancer (NSCLC) cell lines, C-PC (3–15 µM, 12–24 h) elevates the LC3-II/LC3-I ratio, reduces p62, and enhances autophagic flux, which is associated with decreased cell viability [86].

Collectively, Spirulina and its bioactive compounds induce apoptosis and autophagy through multiple complementary mechanisms. However, most evidence is derived from preclinical studies, and clinical investigations are needed to confirm their efficacy and safety in humans.

### 5.3. Cell Cycle Disruption and Arrest

**Figure 7:**
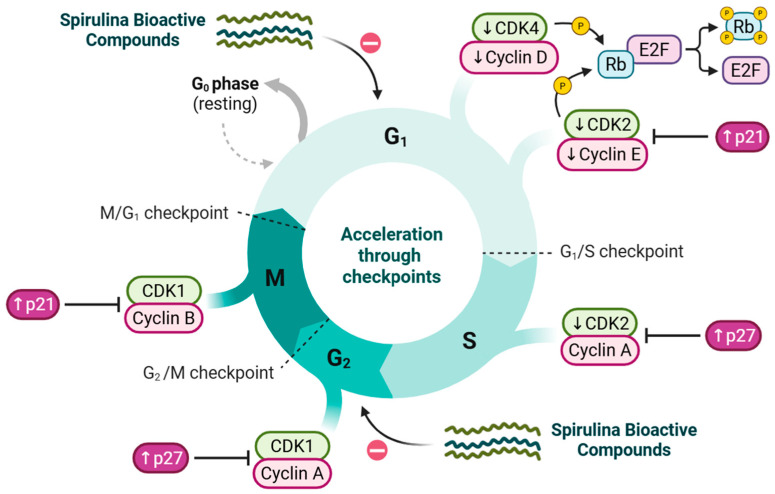
Cell cycle regulation by Spirulina bioactive compounds in cancer cells. Spirulina compounds disrupt cell cycle progression at multiple checkpoints. G0/G1: ↓ cyclin D/CDK4, ↑ p21; G1/S: ↓ cyclin E/CDK2, ↑ p21, ↓ Rb phosphorylation → inhibition of E2F-mediated S-phase entry; G2/M: ↑ p27 inhibits cyclin A/CDK1, preventing mitotic entry. These mechanisms suppress proliferation and sensitize cancer cells to apoptosis.

Spirulina bioactive compounds suppress cancer cell proliferation by inducing cell cycle arrest at critical checkpoints, thereby halting uncontrolled growth in solid tumors (Figure 7). In TGF-β–induced cervical cancer Caski cells, treatment with C-PC (300 µg/mL, 24 h) was associated with G0/G1 phase arrest, mechanistically linked to the degradation of cyclin D1 [88]. In NSCLC tumors, treatment with Spirulina water extract (100–250 µg/mL, 24 h) predominantly induced G1/S checkpoint arrest, driven by a 40–52% reduction in cyclin D1 expression and inhibition of CDK4 activity, along with hypo-phosphorylation of Rb and Akt proteins [17]. This consequently prevents E2F release and halts S-phase progression [89].

Similarly, in breast cancer cells, C-PC (5 µM, 24 h) downregulated cyclin E and CDK2 by 1.60- and 1.64-fold, respectively, while upregulating the tumor suppressor p21 by 1.81-fold, effectively inducing cell cycle arrest at the G1/S transition [77]. Spirulina has also been reported to arrest NSCLC, pancreatic, and ovarian cancer cells in the G2 phase, preventing their entry into the M phase, while having no effect on the cell cycle progression of normal HFF fibroblasts [87,90,91].

Moreover, in human erythro-myeloid leukemia (K562) cells, C-PC (IC 50 = 128.6 µM, 12–16 h) exerts potent antiproliferative effects by inhibiting BCR-ABL signaling and its downstream PI3K/AKT pathway [92]. Phenotype-based experiments combined with network analysis have also demonstrated that C-PC can suppress melanoma cell proliferation through downregulation of the GRB2/ERK signaling pathway [93].

Collectively, these findings indicate that Spirulina bioactive compounds not only prime cells for downstream apoptosis but also induce cell cycle arrest at multiple checkpoints, thereby enhancing their overall anticancer potential. Nonetheless, in vivo studies are essential to confirm these effects and to fully elucidate the underlying mechanisms.

### 5.4. Relation of Spirulina Anti-Angiogenic Activity to VEGF-VEGFR2 Pathway Inhibition

Angiogenesis, the growth of new blood vessels, is crucial for normal physiology but becomes dysregulated in cancer, promoting tumor progression and metastasis through an imbalance of pro- and anti-angiogenic factors [94]. Spirulina exhibits potent anti-angiogenic effects through multiple complementary mechanisms. In vitro, Spirulina-derived C-PC (3 µM, 24 h) suppresses VEGFR2 transcription by 1.17-fold in triple-negative breast cancer cells [77] and may additionally interfere with VEGFR1-mediated signaling, as supported by molecular docking analyses [95]. Moreover, molecular docking studies indicate that the Spirulina pigment phycocyanobilin can directly bind VEGF-A, preventing its interaction with VEGFR2 and thereby inhibiting downstream angiogenic signaling [96].

In vivo, Spirulina administration (250–500 mg/kg from week 25 to 28) enhances tumor control and induces anti-angiogenic effects through modulation of apoptosis-related proteins, notably Bax and p53 in hepatocellular carcinoma [97]. Similarly, C-PC (200 mg/kg for 18 weeks) inhibits HIF-1α, a key regulator of hypoxia-driven tumor angiogenesis, in DMH-induced rat models [95]. Spirulina also reduces endothelial cell migration and tube formation and decreases metalloproteinase activity, including MMP-2, MMP-9, and MCP-1, contributing to its anti-angiogenic profile [95,98].

Interestingly, in Spirulina-treated mice (0.5 g/kg/day for 2 weeks), pancreatic tumors showed a marked reduction in CD31 staining, indicating suppressed vascularization, despite a substantial increase in VEGF-A levels (up to 50%) and a twofold rise in VEGF-A mRNA [98]. This apparent paradox highlights that elevated VEGF alone is insufficient to restore functional angiogenesis, which requires coordinated activation of VEGF receptors and downstream endothelial signaling. Spirulina’s anti-angiogenic activity may therefore persist despite compensatory VEGF upregulation, potentially due to receptor downregulation, impaired endothelial responsiveness, or disruption of VEGF receptor signaling. Furthermore, metabolomic analyses have revealed that several Spirulina metabolites exhibit high structural similarity to CHEMBL3559503, a synthetic immunomodulatory oligonucleotide with established anti-angiogenic activity mediated through inhibition of the VEGF/VEGFR2 pathway [99]. Additionally, a PSP extract (100 µg/mL, 72 h) from *Spirulina platensis* has been shown to inhibit proliferation, migration, and tube formation of vascular endothelial cells (HUVEC) in vitro, accompanied by reduced phosphorylated AKT and ERK1/2, two key kinases in angiogenic signaling [100]. Collectively, these observations indicate that Spirulina can target multiple nodes of angiogenic regulation, effectively suppressing endothelial activation and vessel formation even in the presence of elevated VEGF levels.

Nevertheless, sustained VEGF overproduction could contribute to adaptive resistance mechanisms or rebound angiogenesis if inhibitory pressure on the VEGF signaling axis is relieved. Tumors can compensate for VEGF pathway inhibition by activating alternative pro-angiogenic signals such as PlGF, FGF2, PDGF-B, or Ephrin ligands [101]. These compensatory responses, widely reported in anti-VEGF therapies, underscore the complexity of angiogenic regulation and the potential need for multi-target therapeutic strategies [102].

Although the anti-angiogenic potential of C-PC and other Spirulina-derived compounds appears promising, current evidence is largely limited to in vitro and preclinical studies, and comprehensive clinical investigations are required to confirm their efficacy, safety, and potential integration into combination therapies targeting multiple angiogenic signaling networks.

### 5.5. Metastasis Suppression Through EMT Inhibition and Invasion Prevention Activity

Cancer metastasis is attributed to the mobility of cancer cells and their ability to infiltrate blood vessels and invade adjacent tissues [103]. Spirulina-derived compounds interfere with multiple steps of the metastatic cascade through several mechanisms, including downregulation of MMP-2 and MMP-9 expression [104,105], suppression of MCP-1 and COX-2 [95], and enhancement of cell–matrix adhesion [77].

Epithelial-to-mesenchymal transition (EMT) facilitates metastasis by reducing cell–cell adhesion and increasing cellular motility [106]. In this regard, it was shown that C-PC (0.5–1 mg/mL, 48 h) downregulates EMT-associated regulators such as vimentin, TMEFF2, TGFβRI, Smad4, Snail, Slug, Twist1/2, and ZEB1, correlating with reduced metastatic potential in endometrial cancer xenografts [107]. C-PC (40 μM, 48–72 h) also inhibits migration, invasion, and metastasis of pancreatic cancer cells by increasing E-cadherin and reducing vimentin, fibronectin, ICAM-1, VCAM-1, and MMP-9 via the Akt/β-catenin pathway [108].

Finally, C-PC (7.5 µM, 72 h) has been further shown to inhibit migration in NSCLC cells through downregulation of RIPK1/NF-κB signaling, reinforcing its anti-metastatic potential [109].

**Figure 8:**
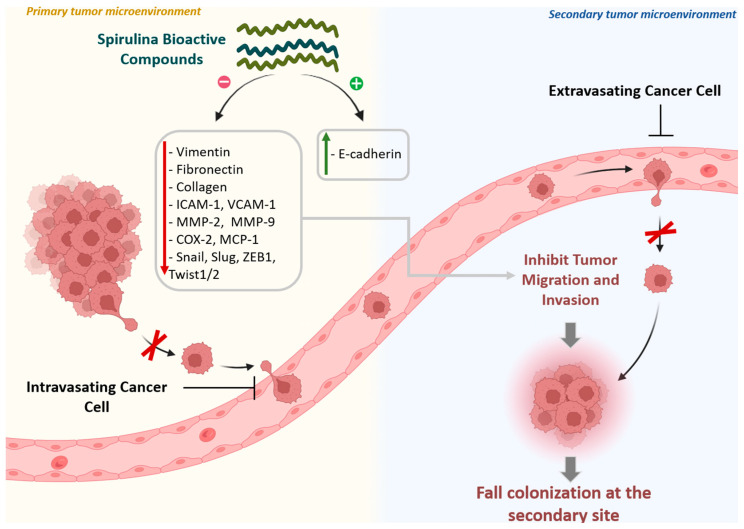
Anti-metastatic actions of Spirulina bioactive compounds. Spirulina compounds inhibit cancer cell migration and invasion by downregulating vimentin, Fibronectin, collagen, MMP-2/9, COX-2, MCP-1, ICAM-1, VCAM-1 and EMT regulators (Snail, Slug, ZEB1, Twist1/2) while increasing E-cadherin. They also enhance cell–matrix adhesion, limiting circulation and colonization at secondary sites.

Collectively, C-PC derived from Spirulina demonstrates multi-targeted inhibition of EMT and cancer cell invasion, highlighting its potential as an adjunctive anti-metastatic therapy (Figure 8).

The pharmacological actions and molecular mechanisms described in Section 5 of Spirulina-derived compounds are summarized in Table 1, providing an overview of their anticancer effect.

## 6. Enhancing Anti-Tumor Immunity Owing to the Immunomodulatory Properties of Spirulina

### 6.1. Macrophage Polarization and Innate Immunity Enhancement

Macrophage polarization is central to anti-tumor immunity, with M1 macrophages supporting tumoricidal effects, while M2 macrophages enhance tumor progression [110]. The Spirulina-derived PSP (500–1500 mg/kg/day, for 15 days) reportedly induces a tumoricidal M1 macrophage phenotype in vivo, engaging TLR4 on macrophages and triggering downstream activation of NF-κB and STAT1 [51]. Complementary signaling pathways, such as MAPK activation, further drive the transcription of pro-inflammatory and immune-stimulatory genes characteristic of M1 polarization [111]. This leads to enhanced production of NO and pro-inflammatory cytokines, including TNF-α and IL-6, as well as strengthened phagocytic and cell cytotoxicity of RAW264.7 macrophages [51], key features of an effective anti-tumor immune response. These effects are supported by an earlier study demonstrating that peritoneal macrophages incubated in vitro with a hot-water extract of Spirulina at 100 µg/mL for 24 h showed enhanced phagocytosis and increased IL-1 production [112]. Recent studies have further highlighted the immunomodulatory potential of Spirulina-derived extracellular vesicles (SPEVs). Following intraperitoneal administration of 20 µg SPEVs per C57BL/6 mouse for 2 days, SPEVs induced sustained neutrophilia (CD11b^+^ Ly6G^+^), increased M1 macrophage infiltration (CD11b^+^ F4/80^+^ CD11c^+^), and reduced cDC1 cells (CD11c^+^ CD103^+^). These changes were followed by the recruitment of IFNγ^+^ Th1 cells (CD3^+^ CD4^+^ CD44^+^), reflecting a shift toward a pro-inflammatory, tumoricidal immune environment [113]. Collectively, these observations indicate that Spirulina-derived compounds can both directly activate innate immune cells and orchestrate adaptive immune responses, positioning them as promising adjuncts in cancer immunotherapy.

### 6.2. Activation of Natural Killer Cells and Enhancement of Cytotoxicity

NK cells are key to innate immunity and cancer immunosurveillance and exert their effects independent of antigen presentation [114]. Notably, a study reported that supplementation with Immulina^®^ (Nordic Immotech A/S, Denmark), a Spirulina-derived PSP (400 mg/day for 7 days in healthy North American individuals), led to a 40% increase in the killing of K562 human erythroleukemic tumor cells by NK cells. The same study also observed elevated mRNA expression of the NK cell marker NKG2D and the cytotoxic effector perforin [115]. Moreover, treatment with Spirulina (0.5 and 1 mg/mL, 48 h) has been shown to enhance NK cell proliferation, density, and overall cell-mediated cytotoxicity [50]. Spirulina (1 mg/g, for 3 weeks) can also enhance NK cell activity indirectly through cytokine production by antigen-presenting cells in vivo. Mechanistically, NK cell function is promoted via MyD88-dependent IL-12 and IL-18 production by DCs and macrophages, mediated through the NF-κB and MAPK pathways, which leads to increased pro-inflammatory cytokine expression [116]. In healthy volunteers, oral administration of a hot-water Spirulina extract (50 mL/day, for several weeks or months) activated macrophages and monocytes via TLR2 and TLR4, resulting in elevated cytokine levels that support NK cell proliferation, cytotoxicity, and IFN-γ secretion [117]. Furthermore, in mice, the PSP fraction (~10 mg/day, for 4 or 5 days) specifically activated monocytes via CD14 and TLR2, but not TLR4 [52]. Collectively, these findings underscore Spirulina’s ability to enhance NK cell-mediated cytotoxicity via both direct activation and cytokine-dependent mechanisms, highlighting its potential as an immunomodulatory agent in cancer therapy.

### 6.3. T-Cell Response Modulation and Adaptive Immunity

T-cells regulate the extent and magnitude of anti-tumor immunity, with CD8^+^ cytotoxic T lymphocytes responsible for engaging directly with and eliminating tumor cells, while CD4^+^ T cells regulate immune responses [118]. Spirulina LPS (20 µg/day for 22 days) skews T-cell differentiation toward a Th1 phenotype at the expense of Th17 cells, via IL-12/STAT4 upregulation and IL-23 suppression [58]. This shift reduces RORγt and IL-17 levels, enhances the expression of IFN-γ that supports M1 macrophage activation, and reduces IL-10 production, which alleviates tumor-associated immunosuppression [119,120]. Insofar as modulation of T-cell activity involves selective suppression of Treg function and sparing effector T-cell function [121]. A recent study demonstrated that Spirulina (50 mg/kg/day for 56 days) reduced Foxp3^+^ Treg frequencies while markedly increasing CD4^+^/CD127^+^ effector memory T cells [122]. Functionally, this involves direct effects on the survival and function of Treg by regulating the IL-2 signaling pathway, along with inhibition of TGF-β production and enhanced effector T-cell resistance to Treg-mediated suppression [123]. The enhancement in CD8^+^ T-cells includes enriched cytotoxic activity, along with induction of memory formation and tumor infiltration [124]. In healthy male volunteers, Spirulina treatment (200 mg/day for 56 days) increased CD4^+^ T-helper cell proliferation and elevated Th1-type cytokine production (TNF-α, IL-2, IFN-γ), while partially inhibiting Th2 cytokines such as IL-4, with no effect on IL-10 [122]. Collectively, these findings underscore the immunomodulatory potential of Spirulina and its derivatives, positioning it as a promising adjunct in cancer immunotherapy.

## 7. Chemoprotective and Adjuvant Therapeutic Applications of Spirulina

### 7.1. Hematopoietic Protection and Immune Recovery

Spirulina treatment was shown to be beneficial in reducing chemotherapy-induced myelosuppression and supporting immune recovery in cancer patients [123,125]. This was highlighted by the findings that Spirulina supplementation (3 × 100 mg capsules, three times daily) during the first two cycles of chemotherapy was associated with significant increases in CD8^+^ T cell counts and IgM levels, which in turn led to a notable decrease in the incidence of severe neutropenia and treatment delays [123]. This aspect of Spirulina is particularly important because myelosuppression is the most common dose-limiting toxicity for most patients undergoing chemotherapy, often resulting in treatment delays [126]. Preclinical studies support the notion that Spirulina can mitigate the bone marrow toxicity in cancer patients undergoing treatment. In cyclophosphamide (Cy)-treated mice, Spirulina-derived PSP (50–1500 mg/kg/day for 10 and 14 days) restored hematopoiesis, increased bone marrow cellularity and blood cell recovery, upregulated cytokines including IL-1, IL-3, GM-CSF, and TNF-α, and improved immune organ function, as indicated by higher thymus and spleen indices, peripheral blood lymphocytes, and white blood cell counts [51,127]. Functionally, these effects were demonstrated to involve the upregulation of Bcl-2 and the activation of NF-κB and STAT pathways, resulting in enhanced cytokine transcription [127,128].

This highlights the ability of Spirulina to support immune reconstitution, reduce the risk of infection, and improve tolerance to chemotherapy [129].

### 7.2. Hepatoprotection Against Drug-Induced Liver Injury

In addition to its immunomodulatory effects, Spirulina’s C-PC mitigated carbon tetrachloride (CCl_4_)-induced liver injury in vitro (31–250 μg/mL, 48 h) and in vivo (75 mg/kg/day) by lowering ROS and MDA levels and restoring SOD, CAT, GPx, and GSH activity [130,131]. C-PC also prevented CCl_4_-induced increases in transforming growth factor-β1 (TGF-β1) and hepatocyte growth factor (HGF) expression, highlighting its antioxidant and anti-inflammatory effects [130]. Spirulina pre-treatment (500 mg/kg/day, for 21 days) was found to attenuate methotrexate (MTX)-induced liver toxicity and immunosuppression by reducing lipid peroxidation and decreasing pro-apoptotic markers (caspase-3 and Bax), thereby preserving the architecture of splenic and hepatic tissues [132]. This was in agreement with a recent report documenting the capacity of Spirulina (500 mg/kg/day, for 21 days) during MTX therapy to enhance hepatic SOD, CAT, and GSH levels, while reducing MDA concentrations compared with MTX alone [133].

### 7.3. Nephroprotection and Renal Function Preservation

In addition to its anticancer properties, Spirulina exhibits significant nephroprotective effects, observed in cisplatin-induced toxicity [134], which can transition to acute or chronic renal injury [135]. Its protective actions involve attenuation of oxidative stress in renal tubules, downregulation of proinflammatory mediators, and preservation of renal blood flow [134]. Experimental models have demonstrated that Spirulina supplementation normalizes plasma creatinine, reduces urea serum levels, and minimizes tubular necrosis [134]. Spirulina exerts these effects by increasing antioxidant enzyme activities, including SOD and CAT, suppressing lipid peroxidation, and reducing apoptosis in renal tissues [136]. Additionally, Spirulina protects against γ-irradiation- and thioacetamide-induced nephrotoxicity by modulating miR-1/miR-146a and reducing oxidative stress and apoptosis through regulation of the AMPK/mTOR pathway [137].

### 7.4. Spirulina’s Neuroprotection and Cognitive Function Preservation

Spirulina has demonstrated significant neuroprotective effects against cognitive injuries in animal models [138]. In these studies, C-PC (50 mg/kg/day, for 3 weeks) reduced oxidative stress and neuroinflammation, protected mitochondrial function, and preserved the structure of hippocampal synapses in doxorubicin-treated models [139]. Treatment with Spirulina (50 and 100 mg/kg/day, for 16 days) was also shown to decrease microglial activation by lowering pro-inflammatory cytokine levels, reduce brain MDA accumulation, and increase antioxidant enzyme activity, thereby supporting mitochondrial function and maintaining neuronal integrity [140]. Behavioral assessments showed that the offspring of Sprague Dawley rats treated with C-PC (400 mg/kg/day, from conception through lactation) displayed improved spatial, recognition, and working memory, along with reduced anxiety-like behaviors [141]. The mechanisms seem to involve the upregulation of Brain-Derived Neurotrophic Factor (BDNF) and activation of the Cyclic AMP Response Element Binding protein (CREB) signaling pathway [142], collectively supporting neural resilience during chemotherapy-induced oxidative and inflammatory stress.

## 8. Clinical Limitations and Regulatory Considerations of Spirulina Use in Oncology

### 8.1. Safety Profile and Adverse Event Considerations

Spirulina has a well-established safety profile, supported by toxicological studies that show no acute or chronic toxicity at high doses in animal models [143,144]. The U.S. FDA recognizes Spirulina as GRAS (GRN 127) for doses of up to 3 g/day [20]. Adverse events are generally mild and rare, including abdominal discomfort, allergic reactions, or skin irritation, often linked to C-PC or impurities introduced during cultivation and processing [56,145,146].

Nevertheless, the safety of Spirulina products may also depend on cultivation conditions and environmental contamination, as cyanobacteria are capable of adsorbing heavy metals from their surroundings [147]. Previous studies have shown that *Spirulina platensis* possesses a strong sorption capacity for certain metals such as cadmium, which may influence the elemental composition of derived products [148]. Analytical assessments have detected trace levels of lead, mercury, cadmium, and arsenic in some Spirulina preparations, although concentrations vary depending on cultivation source, dominant cyanobacterial species, and purification processes [149]. These findings highlight the importance of rigorous quality control and standardized cultivation conditions to ensure product safety. 

Certain populations may also require caution when consuming Spirulina. Individuals with autoimmune disorders, phenylketonuria, or those undergoing immunosuppressive therapy may exhibit increased sensitivity [150,151,152]. In addition, use during pregnancy should be approached carefully, as a small prospective study suggested a possible reduction in fertilization rates among women undergoing in vitro fertilization [153].

### 8.2. Pharmacokinetic and Bioavailability Limitations

A major challenge in the therapeutic use of Spirulina is its low bioavailability, influenced by multiple factors, reducing systemic exposure to active compounds. Structurally, its rigid peptidoglycan cell wall and stable protein-pigment complexes hinder the release and absorption of active compounds, while many are further degraded by gastrointestinal enzymes [154].

Consistent with these limitations, pharmacokinetic analyses in animal models indicate limited systemic availability of key Spirulina-derived compounds. In particular, C-PC, which is metabolized into its bioactive chromophore phycocyanobilin (PCB), shows low systemic exposure following oral administration. In mice receiving a single oral dose of 50 mg/kg, PCB reached a maximum plasma concentration (Cmax) of 40.19 ± 6.35 ng/mL at a rapid Tmax of 0.04 ± 0.01 h, with an elimination half-life (t½) of 3.36 ± 0.83 h and a total systemic exposure (AUC0–last) of 27.27 ± 2.78 h·ng/mL [155]. These pharmacokinetic parameters further support the limited bioavailability of Spirulina-derived compounds after oral intake.

To overcome these constraints, several enhancement strategies are currently being developed and tested. These include enteric coating formulations for protecting pH-sensitive compounds, nanoparticle delivery systems, co-administration with absorption enhancers (such as piperine and quercetin), and lipid-based formulations aimed at increasing solubility and intestinal absorption [156,157]. These and other limitations partly explain the limited number of clinical trials investigating Spirulina’s role in oncology.

### 8.3. Limited Clinical Evidence and Study Design Issues

The only trial assessing the clinical potential of Spirulina was conducted in India among tobacco chewers with oral leukoplakia [158]. Results obtained showed that a daily supplement of *Spirulina fusiformis* resulted in complete lesion regression in 45% of participants, compared to 7% in the control group [158]. Clinical evidence supporting a future role for Spirulina in oncology remains limited despite the initial success [56]. Other trials on Spirulina’s role in cancer therapy are constrained by the relatively small sample sizes, inconsistent dosing regimens and formulations, short follow-up durations, and a lack of clinical outcomes [56]. It is noteworthy that regulatory approval for clinical evaluation of Spirulina requires an IND (Investigational New Drug) application, along with a preclinical toxicology profile and a clinical development plan that aligns with agency expectations. However, relying on this single, 30-year-old study to support the modern translational potential of Spirulina remains insufficient [158]. Over the past three decades, no large-scale clinical trials have successfully advanced despite promising preclinical findings. This translational gap likely reflects several challenges, including difficulties in standardizing Spirulina preparations and defining consistent dosing regimens, heterogeneity in early study designs, regulatory barriers, and limited investment in rigorous clinical development for natural products. Consequently, although preclinical evidence remains encouraging, robust and well-designed contemporary clinical trials are required to more definitively evaluate the therapeutic potential of Spirulina in oncology.

## 9. Conclusions and Future Directions

Spirulina-derived bioactive compounds exhibit diverse pharmacological effects, modulating apoptosis, oxidative stress, metastasis, angiogenesis, and immune responses, while also providing protection against treatment-induced toxicity. Despite these promising properties, clinical translation remains limited by variability in extraction methods, heterogeneous chemical composition, low bioavailability, and scarce clinical validation. Rather than acting as a single therapeutic agent, Spirulina represents a complex reservoir of bioactive molecules capable of targeting multiple complementary pathways involved in tumor progression. Future research should therefore prioritize the systematic identification and characterization of key bioactive constituents and clarification of their complementary and synergistic mechanisms. In parallel, optimizing formulations, delivery systems, and dosing strategies is essential to improve efficacy and reproducibility. Ultimately, well-designed preclinical and clinical studies will be required to validate safety and therapeutic efficacy, paving the way for the rational integration of Spirulina-derived compounds as multi-target adjuncts in modern cancer therapy.

## Figures and Tables

**Table 1:** Pharmacological Activities and Anticancer Mechanisms of Spirulina-Derived Compounds in Tumor Models.

Compound	Tumor Model	Effect	Molecular Target/Pathway	Ref
Gallic acid (52.9 µg/mL), Vanillic acid (14.58 µg/mL), Syringic acid (12.5 µg/mL), Ferulic acid (74.5 µg/mL), Cinnamic acid (36.9 µg/mL) (for 72 h)	Esophageal adenocarcinoma (SK-GT-4)	Pro-oxidant; apoptosis induction	ROS ↑, DNA damage	[65,67,68]
C-PC (30 µM, 6–24 h)	Rat histiocytic tumor (AK-5)		Intracellular ROS ↑, Bcl-2 ↓, caspase-3 ↑	
GM15 peptide (25 μM, 4 h)	Oral cancer (KB)		Intracellular ROS ↑, caspase-9 ↑, DNA degradation	
GM15 peptide (6.25 μM, 4 h)	Oral cancer (KB)	Antioxidant; DNA degradation	Oxidative stress ↓	[68]
Chlorophyllin (125 μM, 24 h)/Phycocyanobilin (60 µΜ, 24 h)	Pancreatic cancer cells (PA-TU-8902)	Antioxidant; inhibits tumor growth	Intramitochondrial ROS ↓, HMOX ↑	[18]
C-PC (100 µg/mL, 24 h)	Glioblastoma (U87/U87-EGFRvIII)	Antioxidant; Context-dependent apoptosis; chemo sensitization	ROS ↓, catalase ↑, MnSOD ↑	[72]
C-PC (5–50 µM, 6–48 h)	Triple-negative breast cancer cells (MDA-MB-231), chronic myeloid leukemia cells (K562) prostate cancer cells (LNCaP)	Intrinsic apoptosis pathway	Bax ↑, Bcl-2 ↓, cytochrome c release, caspase-9 ↑,	[77,78,79]
C-PC (50–200 µg/mL, 24 h)	Triple-negative breast cancer cells (MDA-MB-231), Cervical cancer cells (Hela)	Extrinsic apoptosis pathway	Fas/CD95 ↑	[81,82]
C-PC (10 µM, 48 h)	Pancreatic cancer cells (PANC-1)	Induces autophagic cell death	Beclin-1 ↑, LC3-II ↑, PI3K/Akt/mTOR ↓, NF-κB ↑	[86,87]
C-PC (3–15 µM, 12–24 h)	Non-Small-Cell Lung cancer cells (H460, A549, LTEP-a2 and H1299)		LC3-II/LC3-I ↑, p62 ↓	
C-PC (300 µg/mL, 24 h)	Cervical cancer (Caski)	G0/G1 arrest; cell cycle inhibition	Cyclin D1 ↓	[88]
C-PC (5 µM, 24 h)	Triple-negative breast cancer cells (MDA-MB-231)	G1/S arrest; cell cycle inhibition	Cyclin E ↓, CDK2 ↓, p21 ↑	[77]
C-PC (IC50 128.6 µM, 12–16 h)	Erythro-myeloid leukemia (K562)	Antiproliferative	BCR-ABL/PI3K/AKT ↓	[92,93]
C-PC (6 µM, 24 h)	Melanoma cells (A375, M14)		GRB2/ERK ↓	
C-PC (3 µM, 24 h)	Triple-negative breast cancer cells (MDA-MB-231)	Anti-angiogenic	VEGFR2 ↓, Interferes with VEGFR1-mediated signaling	[77,95]
C-PC (200 mg/kg, 18 weeks)	DMH-induced rat model		HIF-1α ↓	
C-PC (0.5–1 mg/mL, 48 h)	Endometrial cancer xenograft	Anti-metastatic	EMT regulators ↓ (vimentin, Snail, Slug, Twist1/2, ZEB1)	[107,108,109]
C-PC (40 µM, 48–72 h)	Pancreatic cancer cells (PANC-1 and BxPC3)		E-cadherin ↑, vimentin/fibronectin/ICAM-1/VCAM-1/MMP-9 ↓; Akt/β-catenin pathway ↓	
C-PC (7.5 µM, 72 h)	NSCLC cells (H358, H1650, and LTEP-a2)		RIPK1/NF-κB ↓	

## Data Availability

No new data were created or analyzed in this study.
